# Bearing Fault Diagnosis Based on the Switchable Normalization SSGAN with 1-D Representation of Vibration Signals as Input

**DOI:** 10.3390/s19092000

**Published:** 2019-04-29

**Authors:** Dongdong Zhao, Feng Liu, He Meng

**Affiliations:** Research Center for High-Speed Railway Network Management of Ministry of Education, School of Computer and Information Technology, Beijing Jiaotong University, Beijing 100044, China; ddzhao@bjtu.edu.cn (D.Z.); hemeng@bjtu.edu.cn (H.M.)

**Keywords:** bearing, fault diagnosis, GAN, semi-supervised

## Abstract

The bearing is a component of the support shaft that guides the rotational movement of the shaft, widely used in the mechanical industry and also called a mechanical joint. In bearing fault diagnosis, the accuracy much depends on the feature extraction, which always needs a lot of training samples and classification in the commonly used methods. Neural networks are good at latent feature extraction and fault classification, however, they have problems with instability and over-fitting, and more labeled samples must be trained. Switchable normalization and semi-supervised learning are introduced to solve the above obstacles in this paper, which proposes a novel bearing fault diagnosis method based on switchable normalization semi-supervised generative adversarial networks (SN-SSGAN) with 1-dimensional representation of vibration signals as input. Experimental results showed that the proposed method has a desirable 99.93% classification accuracy in the case of less labeled data from the public data set of West Reserve University, which is better than the state-of-the-art methods.

## 1. Introduction

In the past, fault diagnosis of the bearing component has mainly relied on listening sticks to contact the bearing component, which is extremely demanding for the technician who is responsible for hearing the difference between the norm and failure. Also, technical staff may judge differently in the same situation. Gradually, electronic stethoscopes have replaced clumsy listening sticks, increasing the sensitivity of the diagnosis. Following on from this, researchers found that the vibration frequency of the bearing is inconsistent compared with a normal signal, and the vibration frequency characteristics formed by different faults are different. Therefore, various acceleration sensors and vibration measuring instruments were introduced to collect vibration data. The use of this data, the collection of device data, and the analysis of large data by algorithms also improved the diagnostic ability to some extent. Recently, experts in the field of bearing fault diagnosis technology have paid close attention to the inherent causes of faults. Therefore, they began to capture the intrinsic characteristics of the collected data through the intelligent algorithms to achieve fault diagnosis. Among the diagnostic methods, the most commonly used are: Fast Fourier Transform [1,2], Fuzzy Mathematics [3,4,5,6], Grey System Theory [7,8], Wavelet Transform [9,10,11,12,13,14,15,16,17,18], Expert System [19,20,21], SVM [22], and Artificial Neural Networks [19,20,21,22,23,24,25,26,27,28,29,30,31,32,33,34,35]. From the perspective of data processing, the current main focus is on two-dimensional and one-dimensional methods. This has greatly improved the level of bearing fault diagnosis. At present, research on bearing faults is widespread in academic and industrial circles, especially research based on artificial neural networks and machine learning, which is a branch of neural networks. Zhang proposed a supervised bearing fault diagnosis method based on convolution Neural Network with Wide first-layer kernels (WDCNN) [29], which includes five convolution layers, a fully-connected layer and a Softmax layer. Then, Li proposed a bearing fault diagnosis method based on a fully-connected winner-take-all auto encoder, which used unsupervised learning to get bearing features by using an auto encoder, supervised fine tuning to optimize the model, and then, a Softmax classifier was used to classify faults, because the loss of the auto encoder is based on the re-built error, which make images more or less fuzzy.

Semi-supervised learning and unsupervised learning have been at the center of recent research. In [29], the ratio between training samples and test samples was 26.4, and some fine-tuning was needed while training can stop immediately when generative adversarial network(GAN) obtains the desirable test accuracy and training accuracy. Hence, more and more algorithm research has focused on semi-supervised learning for a better generalization performance; Also, unsupervised algorithms usually have a higher price, at least for now. Following the idea of reducing the number of training samples while maintaining ideal performance, we consider semi-supervised generative adversarial networks based switchable normalization.

Due to the growth in the maturity of the GAN network [36,37], it has been applied to different fields. In the spur of generator, the discriminator can not only learn intrinsic, potential characteristics, but also can discriminate samples. In 2016, [38] proposed the application of GAN for semi-supervised learning (SSGAN). The intuition exploits the samples generated by G in GAN to boost the performance of image classification tasks by improving generalization. The design of the basic GAN is also not the same for different research backgrounds. This paper is mainly aimed at learning the bearing signal vibration latent features and identifying the bearing vibration signal fault categories in real time to obtain the bearing health status information.

In this paper, we propose a semi-supervised generative adversarial network based on switchable normalization (SN-SSGAN) to learn the latent features of the raw bearing vibration signal and to distinguish the fault categories, which consists of a G and a D. The architecture of the D of the proposed SN-SSGAN is basically similar to WDCNN, except that the switchable normalization replaces the batch normalization and all the pooling layer are cancelled. By comparison with the WDCNN, the experimental results of the proposed SN-SSGAN show that by participating in training, the G helps with bearing fault diagnosis performance. At the same time, the D not only can acquire the features with strong resolution, but also becomes a more sensitive classifier with the G, inspired by a large amount of fake data and real unlabeled data from the training data set. Some experiments showed that the proposed model has considerable value in bearing fault diagnosis, and the model could be introduced into engineering practice to provide field workers with more useful information.

The remainder of this paper is organized as follows. An introduction to GAN and SSGAN is provided in Section 2. The intelligent diagnosis method based on SN-SSGAN is given in Section 3. Several experiments were conducted to evaluate our method by comparing it with some other baseline methods. A discussion of the experimental results is presented in Section 4. We draw conclusions and present the future work in Section 5.

## 2. Theory Background

### 2.1. A Brief Introduction to GAN

GAN [36], proposed in 2014, was originally used as a generative model, which can be used to generate images, audio, etc. The quality of generative objects increases year by year. GAN comes originally from a game, and the learning framework is composed of a generator (*G*) and a discriminator (*D*), which play different roles in the game.

(1) $$\underset{G}{\min}\underset{D}{\max}E_{x:\chi }[\mathrm{logp}(y=1|x,D{)]+E}_{z:P(z)}\left[\log(1-p(y=1\right|G(z),D))]$$

For a given training data set, the purpose of the *G* is to generate samples having the same probability distribution as the training data set. The *D* belongs to the common binary classifier and is mainly responsible for two tasks. Firstly, it is necessary to determine whether the input comes from the real data distribution or the *G*. Secondly, the *D* guides the *G* through the back-propagation gradient to create a more realistic sample, which is the only way for the *G* to optimize its model parameters. During the game, the *G* takes in random noise as input and outputs a $G_{sample}$, which is to be maximized by the *D* that this is the probability of the decision from the real training set.

During the training, the *D* takes in the image of the training set as an input for half of the time, and takes in the image $G_{sample}$ obtained by the *G* as an input for the other half of the time. The *D* is trained to maximize the distance between categories, and to distinguish between the real image from the training set and the fake sample from the *G*. Finally, the training will eventually reach equilibrium—Nash equilibrium. Because this equilibrium is difficult to find, there are many research papers for solving this problem [39,40,41].

Therefore, the *G* should be able to make the generated probability distribution and the real data distribution as close as possible so that the *D* cannot distinguish between real and fake samples. Therefore, in the adversarial process, the *G*’s ability to learn the real data distribution becomes stronger and stronger, and the *D*’s feature learning and discriminative ability is also becoming stronger and stronger. Finally, this research is applied in different real-life scenarios, such as image synthesis, scene synthesis, face synthesis, style migration, image super resolution, and image domain conversion.

### 2.2. Deconvolution, Convolution, Normalization and Activation

After a brief introduction to GAN, deconvolution, convolution, normalization and activation will be discussed below.

#### 2.2.1. Deconvolution and Convolution

Convolution performs a perfect conversion of the input data, and is commonly used to obtain a compact and high-level latent feature that lays a good foundation for separation or distinguishing in the future steps.

The standard neural network structure consists of input layer *x*, output layer *y*, and some hidden layer *h*. Every layer has many units. Usually, every hidden unit $h_{j}$ receives all output from the last hidden layer, which is calculated with the formula of non-linear combination as follows:

(2) $$h_{j}=F\left(b_{j}+\underset{i}{\sum }w_{ij}x_{i}\right)$$

$w_{i}{}_{j}$ is the weight value to control the intensity between input units and hidden units, $b_{j}$ is the bias of hidden units, and *F* is the non-linear function, such as the sigmoid function. Commonly, multi-layer neural networks need a lot of parameters. However, with the rapid development of hardware, the dilemma of a lack of computing resource has been solved. Because the convolution neural network depends on feature sharing principles, every feature map output through channel is created by the same size filter. Compared to standard neural network structures, convolution neural networks depend on fewer model parameters. At the same time, the convolution neural network uses a pooling layer to ensure the translation invariance of image. Meanwhile, the pooling operation broadens the receptive field so as to receive more input. The larger receptive field can be good at learning inner feature representation by deep layer learning. Average pooling, one of the most common operations, averages the pixel value of the receptive field to comprehensively consider the characteristics of surrounding pixels. Max pooling extracts important information from receptive field pixels’ value to void model learning unused features.

Deconvolution and convolution are basically the same, the difference is mainly that deconvolution requires a filling process and needs to be cropped after the last deconvolution. In this paper, the *D* is equivalent to an encoder with a classifier, and the *G* is equivalent to a decoder. The convolution features are usually used as input data for the classifier. Usually, the performance of the classifier depends on not only the data quality of the convolution feature, but also the methods used in the classification, and even the normalization of the intermediate stages. So, normalization methods, activation layers, and classification layers will be introduced next.

#### 2.2.2. Normalization

In order to improve the stability and generalization of model training, there are an increasing number of normalization methods available. Normalization is a special function transformation method for logarithmic values. That is, assuming that there is a normalized function f, the original value before the normalization is converted x, and finally a normalized value f(x) is obtained. The so-called normalization is to satisfy certain characteristics by converting values in order to prevent the entire network from collapsing during training, especially in deep works. The current standardization methods applied to neural networks can currently be divided into three categories:

The first: to normalize the weights on the edges of connected neurons, for example, weight normalization, and this adds L1 regular terms or L2 regular terms to the Loss function to avoid over-fitting of the model during training.

The second: to normalize the activation values of layer neurons, such as Batch Normalization(BN) [42], Layer Normalization(LN) [43], Instance Normalization(IN) [44], Group Normalization(GN) [45], and spectral normalization(SN) [46].

The third: the fusion of the above methods, for example, switchable normalization [47] (2018) proposed switchable normalization to LN, BN, IN, which is the appropriate normalization in each layer by adding 6 weight parameters.

**Figure 1:**
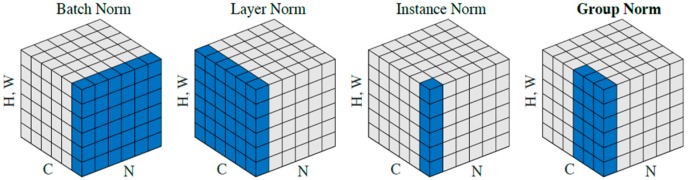
This picture from [45]. Each subplot shows a feature map tensor, with N as the batch axis, C as the channel axis, and (H,W) as the spatial axes. Batch normalization (BN): normalizes the NHW on the batch; Instance normalization (IN): normalizes the HW on the image pixels; Layer normalization (LN): normalizes the CHW in the channel direction; Group Norm groups the channels and then normalize; switchable normalization combines BN, LN, and IN by six weights, and automatically finds a suitable normalization method during training.

The main difference between the methods of recording the input image as [N, C, H, W] is as depicted in Figure 1.

#### 2.2.3. Activation Layer

The activation function is a function that runs on the neurons of the neural network and is responsible for mapping the input of the neurons to the output. In order to improve the ability of the network to express deep features of the input data, a nonlinear activation function is introduced. The commonly used activation layers are sigmoid, tanh, rectified linear unit(ReLU) and leaky rectified linear unit (LReLU).

The sigmoid function, also called the logistic function, is used for hidden layer neuron output, and its value ranges from 0 to 1, which can map a real number to this interval for binary classification.

(3) $$f(x)=\frac{1}{1+\exp(-x)}$$

The tanh function, also a tangent function, ranges from −1 to 1.

(4) $$\tanh(x)=\frac{\sinh(x)}{\cosh(x)}=\frac{e^{x}-e^{-x}}{e^{x}+e^{-x}}$$

The above sigmoid and tanh are saturation activation functions, while *ReLU* and its variants are unsaturated activation functions. The advantage of using the unsaturated activation function is two-fold: first, the unsaturated activation function solves the so-called gradient disappearance problem to a certain extent; and second, it speeds up the convergence. *ReLU* outputs the positive number as it is, and the negative number is directly set to zero. The calculation of the *ReLU* function is performed after convolution, so it is the same as the tanh function and the sigmoid function, and belongs to the non-linear activation function. When the input is negative, *ReLU* is not activated at all, which means that once a negative number is entered, *ReLU* cannot be activated.

The formula of *ReLU* function is as follows:

(5) $$ReLU(x)=\{\begin{array}{cc} x & if\;x>0\\ 0 & if\;x\leq 0 \end{array}$$

In contrast, leaky *ReLU* assigns a non-zero slope to all negative values. The function formula is as follows:

(6) $$LReLU(x_{i})=\{\begin{array}{cc} x_{i} & if\;x_{i}\geq 0\\ \frac{x_{i}}{a_{i}} & if\;x_{i}<0 \end{array}$$

where $a_{i}$ is a fixed parameter, i represents that different channels corresponding to $a_{i}$. The Softmax function is used for multi-class neural network output. The Softmax function will compress each class between 0 and 1 and divide by the output sum. It can actually represent the input probability of every class. The Softmax function is best used at the output layer of the classifier. The function is as follows:

(7) $$\sigma (z{)}_{j}=\frac{e^{z_{j}}}{\sum_{k=1}^{K}e^{z_{k}}}$$

### 2.3. Architecture of SSGAN

Semi-supervised learning is one of the most prominent applications of GAN. For the generative scene, the *D* computes the true and false probability for guiding to train the *G*, and may be discarded after training. However, for the scene of semi-supervised, especially multi-class, the *D* in training provides the probability of data from the generated data of the *G* and the real data. Then, these probabilities send messages back for the *G*’s improvement in learning the features of the real data. So, the *D* and *G* improve with each other. This was shown in [38] where the *G* generated realistic data to boost the *D* to class accurately under semi-supervised learning; Also, the *D* could class accurately to provide feedback to the realistic generated data by the *G*.

The *G*, like a decoder, starts with initializing a random vector with a normal distribution, then maps the vector to a higher dimension by a process like decoding (such as the decoder in VAE [47]), and finally generates fake data similar with the shape of the input data of *D*. Then, the auto-encoding calculates the loss function by comparing the difference between each pixel point between the two pictures, and the loss function is calculated by the adversarial process in the generative adversarial network. Obviously, during the adversarial process, the *G* constantly improves itself to try to gain the trust of the *D* [48].

The *G* usually consists of some deconvolution, normalization and activation layers. The *G*’s input is separately a randomly generated vector, and the shape of its output is the same as the input of the *D*. The *D* of SSGAN is usually not the binary classifier, we assume the input data have *K* categories, in supervised learning, the *D* will be a *K* classifier, and in the unsupervised learning, the *D* will be a binary classifier. The extra one is generated by the *G*. The *D* includes some convolution, normalization and fully-connected layers, which end with a Softmax layer.

**Figure 2:**
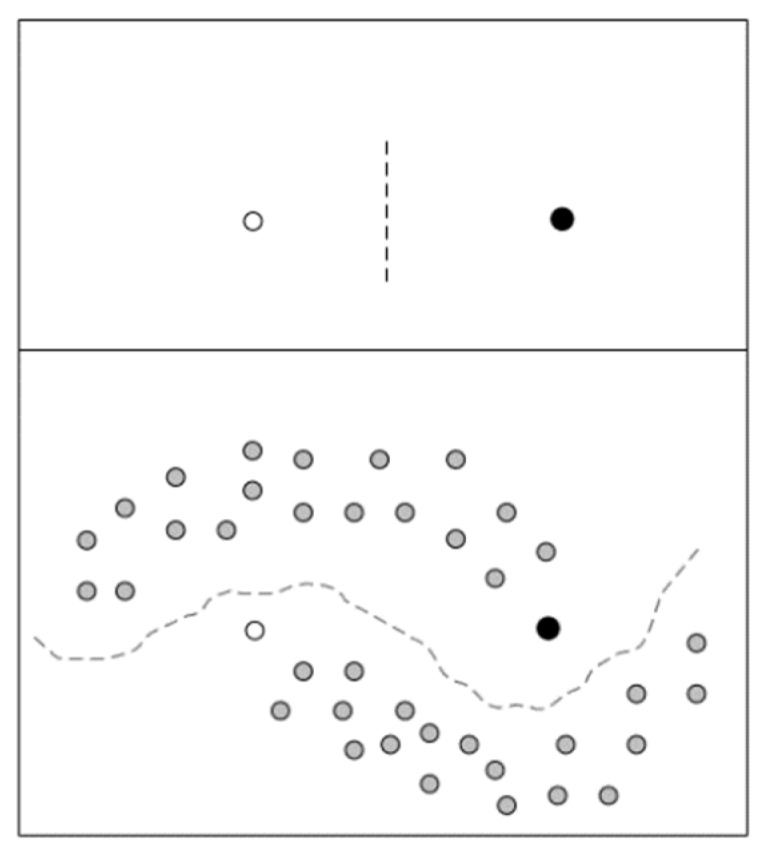
Only samples with labels are shown above; samples with labeled and unlabeled samples are shown below.

In unsupervised learning, fake data and real data are taken into the *D* for the optimization of the *D* to discriminate real and fake data. For the fake data generated by *G*, the *D* tries to judge it as fake data. For the real unlabeled data, the *D* tries to judge it as real data. In semi-supervised GAN, the real data usually consists of labeled data and unlabeled data. Here we need to explain the importance of unlabeled data in semi-supervised learning, as depicted in Figure 2. White and black dots in Figure 2 are different classificatory labeled data, and gray dots are unlabeled data. This figure intuitively conveys the importance of unlabeled data in semi-supervised learning when labeled data are rare.

In the adversarial process, the received data of the *D* is mainly from three points:

(1) Labeled real data: from the training data set. When training, the *D* just needs to try to identify them.
(2) Unlabeled real data: from the training data set. When training, the *D* just needs to regard them as real data, and attempt to give a probability as close to 1 as possible.
(3) Unlabeled fake data: generated by the *G*. When training the *D* tries to distinguish them from unlabeled real data as a probability as close to 0 as possible.

The loss of *D* includes two parts: (1) the loss of unsupervised learning $L_{\mathrm{un}\sup ervised}$, and (2) the loss of supervised learning $L_{\sup ervised}$. So, the total loss *L* is the sum of loss of the supervised and unsupervised learning.

(8) $$L=L_{\sup ervised}+L_{un\sup ervised}$$

Firstly, a standard classifier Softmax should classify a sample data *x* into one of *K* possible categories. The *D* of SSGAN take in x as input and outputs a *K*-dimensional vector of logits $\left\{l_{1},\ldots l_{K}\right\}$, which are finally converted into class probabilities by Softmax: $p_{model}\left(y=j|x\right)=\frac{\exp(l_{j})}{\sum_{k=1}^{K}\exp(l_{k})}$. In supervised learning, SSGAN should be trained by minimizing the cross-entropy between the labels of the real labeled data and SSGAN predictive distribution $p_{\mathrm{mod}el}(y|x)$. In unsupervised learning, the fake data are labeled with a new class y=K+1, then we use $p_{\mathrm{mod}el}(y=K+1|x)$ to represent the probability that *x* is fake data. Assuming the ratio between the fake data and the real data is 1:1,

(9) $$\begin{array}{l} L=-E_{x,y∼P_{data}(x,y)}[\log p_{\mathrm{mod}el}(y|x)-E_{x∼G}[\log p_{\mathrm{mod}el}(y=K+1|x)]]\\ \;=L_{\sup ervised}+L_{un\sup ervised},where\\ L_{\sup ervised}=-E_{x,y∼p_{data}(x,y)}\log p_{\mathrm{mod}el}(y|x,y<K+1)\\ L_{un\sup ervised}=-E\{x∼p_{data}(x)\log[1-p_{\mathrm{mod}el}(y=K+1|x)]+E_{x∼G}\log[p_{\mathrm{mod}el}(y=K+1|x)]\}, \end{array}$$

For unsupervised learning, the *D* outputs true or false. Then we use D(x) to denote $1-p_{\mathrm{mod}el}(y=K+1|x)$:

(10) $$D(x)=1-p_{\mathrm{mod}el}(y=K+1|x)$$

We bring Formula (10) into the $L_{un\sup ervised}$ of Formula (9), then we easily find the formula of the unsupervised loss function $L_{\mathrm{un}\sup ervised}$ of SSGAN is exactly the loss of standard GAN:

(11) $$L_{\mathrm{un}\sup ervised}=-\{E_{x∼p_{data}}{}_{\left(x\right)}\log D(x)+E_{z∼noise}\log(1-D(G(z)))\}$$

After this loss function is determined, training operations start by minimizing this loss. It should be noted that Formulas (9)–(11) come from [38].

## 3. Proposed SN-SSGAN Intelligent Diagnosis Method

In this section, an intelligent method for bearing fault diagnosis will be introduced in detail. Firstly, semi-supervised GAN reduces reliance on observational data sets which is a major downside of WDCNN. Secondly, alongside semi-supervised GAN, switchable normalization is recommended to improve the stability of semi-supervised GAN.

The *D* and *G* of the proposed SN-SSGAN will be detailed in Section 3.1.1 and Section 3.1.2. In this paper, WDCNN is utilized to displace the *D* after some adjustments that delete all pooling layers and replace batch normalization with switchable normalization, which uses wide kernel to extract the characteristic signatures of vibration signals in the intermediate and low frequency bands, and which is easier to train. So, the input shape of *D* is 2048 × 1 which is the same as WDCNN; this means the output shape of *G* is also 2048 × 1. The samples united with 2048 × 1 vibration signals are obtained from raw vibration signals by taking a section of 2048 and slicing with a step length of an overlap size until the end of the vibration signals. Finally, the samples are randomly separated into training samples and test samples.

In the process of supervised learning, training signals with labels are fed into *D*, after some convolution, normalization and activation, the *D* outputs the probabilities of the signal samples. Then the loss is computed according to the gap between the predicted result of the signal samples and its corresponding label, and this optimizes *D*’s parameter.

In the process of unsupervised learning, random vector z are fed into *G*, the *G* outputs the fake data, which are taken in *D* for a probability, these are usually not too close to 1 at the beginning, and the ideal value is 1 or as close as possible, the loss of batch is computed and the next step is to optimize the *G* by fixing the *D*. Then, the training signals that are unlabeled and formed as a batch are also put into *D*. When *D* gives a corresponding score close to 1 this means the data are real Finally, the parameters of *D* are optimized by fixing the *G* for *G* examination in the next round. By some training, the training work is basically over, which means a satisfactory, knowledgeable model is obtained. When the test samples come, the model outputs the fault conditions.

**Figure 3:**
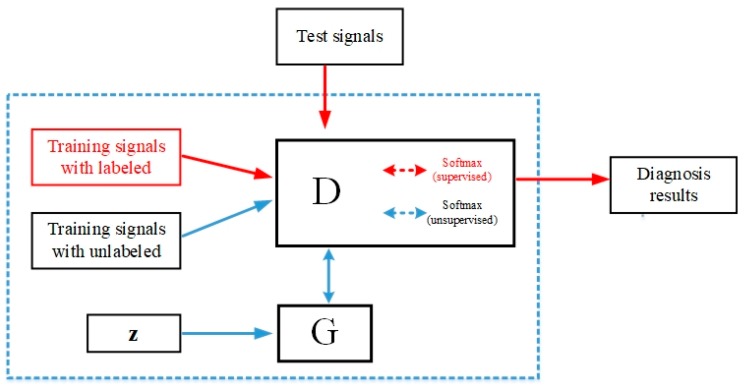
The overall framework of semi-supervised generative adversarial network based on switchable normalization (SN-SSGAN).

Note that supervised learning or unsupervised learning is executed according to whether the coming batch samples have corresponding labels, but because the ratio between labeled samples and unlabeled samples is a specific value, the number of executions of supervised learning and unsupervised learning is also a determined value. The overall framework of the proposed semi-supervised generative adversarial network based on switchable normalization(SN-SSGAN) is shown in Figure 3. The details are introduced in the following subsections.

### 3.1. Architecture of the Proposed SN-SSGAN Model

Semi-supervised learning generative adversarial networks are proposed in [38]. No matter what kinds of variants of GAN exist, there is no limit to the generative and discriminative models of the GAN. The GAN is only a network structure. We can put any generative model and discriminative model into the corresponding location of the GAN. Thus, we can exploit the samples generated by G of SSGAN to improve the performance of image classification tasks by improving generalization. With the development of GAN in recent years, GAN has also experienced some milestone improvements, one of these is deep convolution generative adversarial network(DCGAN), which combines CNN and GAN perfectly. However, in order to improve the quality and convergence speed of the sample, DCGAN makes some changes in the structure of the convolutional neural network, mainly as shown in the following points:

1. Cancel all pooling layers. Use the fractionally stride convolution instead of the pooling layer in the *G* network, and use the stride convolution instead of the pooling layer in the *D* network.
2. Use batch normalization in both *D* and *G*.
3. *ReLU* is used as the activation function in the *G*, and tanh is used in the last layer.
4. *Leaky ReLU* is used as an activation function in the *D*.

**Figure 4:**
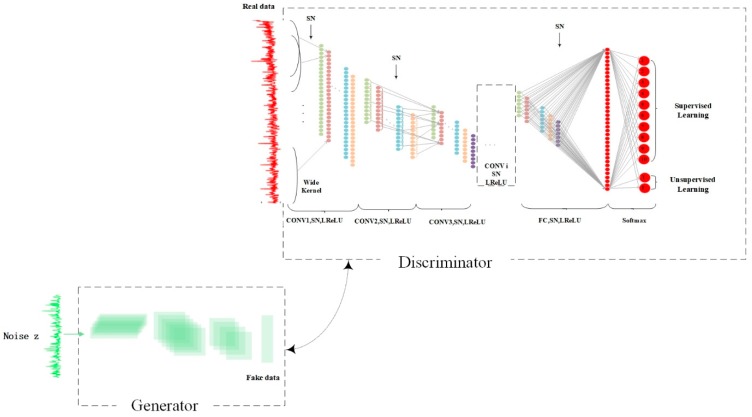
The SN-SSGAN architecture.

Several experiments suggest that it works. The proposed SN-SSGAN in this paper, also used 1, 3 and 4 above. Figure 4 is the proposed SN-SSGAN architecture. In the whole process of SN-SSGAN, there are mainly two parts: supervised learning and unsupervised learning. In the supervised learning, the input of *D* is real labeled data. In the learning stage, the *D* needs to learn the latent feature of the labeled data by a series of convolution, normalization and activation, and finally give a vector of *K* probabilities values where the position value with the highest probability value should correspond to the real label of the data as much as possible. In the unsupervised learning, the input of *D* may be real unlabeled data from the training data set or fake data generated from the *G*. Similarly, the *D* sends learned features to the Softmax classifier, just like a binary classifier that outputs values that are usually either close to 1, which means real data or close to 0, which means fake data. Thus, *D* has judged what is suitable for supervised learning or for unsupervised learning, optimization begins to reduce the error, and then *D* and *G* are alternately trained. More specific parameters about *D* and *G* will be given in Section 3.1.1 and Section 3.1.2.

#### 3.1.1. The Architectural Parameters of the *G*

In the architecture of *G* in the proposed SN-SSGAN, the deconvolution operation is not really a deconvolution, and it is specifically called a fractionally stride convolution. The difference between the fractionally stride convolution and common deconvolution is that the deconvolution adds 0 around the entire input matrix, and the fractionally stride convolution splits the input matrix, adding 0 around each pixel. In order to find a more stable semi-supervised learning model, switchable normalization was introduced to replace batch normalization. The network of *G* in the proposed model has 13 layers in the deep convolutional network with switchable normalization.

The pseudo code is listed as following:

*i.*  input data: data = [BatchSize, z]
*ii.*  data = reshape(data)
*iii.*  data = FC(data)
*iv.*  num = int(ceil(log2(max(h,w))))
*v.*  *i from 0 to (num-2)*
  *1.*  data = deconvolution (data) # the output shape of length/(2^(i+1))
  *2.*  data = SwitchableNormalization (data)
  *3.*  data = ReLU(data)
*vi.*  data = deconvolution (data) # the output shape of length/(2^(num-1)))
*vii.*  data = SwitchableNorma(data)
*viii.*  output data = tanh(data)

Each deconvolution, is followed by switchable normalization and *ReLU* activation. When the random vector *z* is fed into *G*, a series of convolution operations are initiated. In the process from input to output, the shape of the data becomes wider and shallower from deep and narrow. In this paper, we reshape the original bearing vibration data into a one-dimensional (1-D) vector that is fed into the *D*. Therefore, the *G* must also generate one-dimensional vector data for feeding into the *D*. The operation and output shape of every layer is shown in Table 1. The FC indicates fully-connected, and SN is switchable normalization. The operation name and output size are listed in Table 1.

#### 3.1.2. The Architectural Parameters of the *D*

The architecture of the *D* in the proposed SN-SSGAN has 5 convolution layers and one fully-connected hidden layer. The architecture of the *D* is based on the architecture of CNN in WDCNN [29] and deletes the pooling layer, which means the difference between SN-SSGAN and WDCNN lies in the following two points: (1) SN-SSGAN is based on switchable normalization, but WDCNN is based on batch normalization, and (2) SN-SSGAN is an adversarial network, that is, SN-SSGAN has one more *G* than WDCNN. The shape of the input data of *D* is [BatchSize, 2048, 1, 1], and a probability vector is output by Softmax. The specific operation and output size are listed in Table 2.

#### 3.1.3. The Loss Optimization

In order to obtain the global best, not easily fall into the local best, and at the same time improve the training speed in the proposed algorithm, the *D* and *G* are optimized by using AdamOptimizer, and *G* and *D* are each iterated once. In the training, some tricks are used, for example, one-sided label smoothing, and Huber loss.

The *D*’s loss consists of three parts: (1) the loss of real unlabeled data from the training data set, (2) the loss of fake data generated by the *G*, and (3) the loss of supervised learning.

The *G*’s loss consists of two parts: (1) the loss of fake data generated by *G*, and (2) Huber loss weight. The Huber loss is used to obtain a model that has more robustness and is less influence from outliers. The formula to calculate the Huber loss is (12):

(12) $$L_{\delta }(y,f(x))=\{\begin{array}{cc} \frac{1}{2}{\left(y-f(x)\right)}^{2} & if|y-f(x)|\leq \delta \\ \delta *|y-f(x)|-\frac{1}{2}\delta^{2} & else \end{array}$$

Huber loss segments the square loss into square loss and linear loss. When the deviation is less than δ, it is a square loss, and when the deviation is greater than δ, it is a linear loss. Since outliers tend to have large deviations, they fall into the interval of linear loss (less than the original mean square loss), which reduces the degree of punishment for outliers and reduces the impact of outliers on the model.

(13) weight=min(max(0,(s−step)/s),1.0)×10

In Equation (13), s is a preset value, such as 1000, 1500, and step is the training step, which increases as the number of training increases. The multiplication of Huber loss and weight has a certain constraint on the initial training of the *G*. With these solutions, we can safely train the *D* to near optimal, without worrying about the disappearance of the gradient, thus promoting the stability of the training.

### 3.2. Training of SSGAN

**Figure 5:**
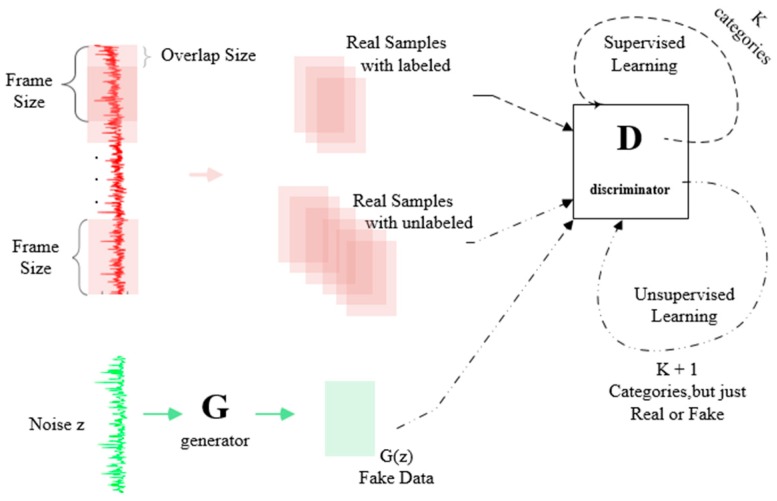
The flow chart of training.

During training, the proposed method minimizes the total loss obtained by directly combining the *G*’s loss and *D*’s loss together. The training steps that will be followed, and the corresponding training flow chart are shown in Figure 5.

1. Initialization: the number of iterations, the labeled samples to participate in the supervised training.
2. To generate fake data: the *G* with input of normal random number vector generates fake data.
3. Classification: fake data, unlabeled real data and labeled data are fed into *D*, obtaining the corresponding discriminant result.
4. Compute the total loss: supervised learning if there is labeled data, unsupervised learning with unlabeled real data and fake data, and finally, compute the *G*/*D* loss.
5. Optimization: fixed *G/D*, to optimize and update the parameters of the *D/G*.
6. Whether to save the model.
7. Iterative training: repeat steps 2–6 until the max step, stop training.

In the training process, some useful training tricks are also applied in the experiment for stable training, such as one-sided label smoothing, gradient clipping, reconstruction loss with an annealed weight, and Adam optimizer with a higher momentum [38], which boost convergence.

In the testing process, the data processing and batch size are the same as training, and then used to test under the same batch size based on the saved model and to average the accuracy of a batch as the final result.

### 3.3. Stable Switchable Normalization

The authors of [49] proposed a new method of switchable normalization to complete a learning-to-normalization, which solves two difficulties as follows:

1. Manually set normalized layer: not a universal method when solving practical problems.
2. Deep neural networks usually have many layers. These normalized layers use only the same normalization operation, because manually designing operations for each normalized layer requires a lot of experiments.

So, switchable normalization can determine the appropriate normalization operation for each normalization layer in a deep network, possibly BN, IN, LN, or their previous combination. That is, SN unifies normalization methods by differential learning so that not only the normalization operation can be performed simultaneously with the optimization of the network parameter, but also to ensure optimization efficiency while maintaining high performance. The result of the experiment comparing switchable normalization and batch normalization with the same parameters is presented in Section 4.3.

## 4. Experiment and Discussion

### 4.1. Data Description

In order to verify the effectiveness of the proposed method, this paper uses bearing data from the US West Reserve University [16]. The data from 12k Drive End Bearing Fault Data includes load 1, load 2 and load 3 for each fault type, and the Normal Baseline Data. So, the data set spans ten fault categories. The data description is shown in Table 3. Training samples and test samples are 14,100 and 3520, respectively. All the data are overlapped from raw data, and the frame size is 2048, and the overlap size is 256. It is important to note that the samples used to train in the experiment vary according to the ratio of labeled samples in all training samples. That is, even when the ratio of the unlabeled data is 1, the ratio between training samples and test samples is only 4. In [29], the ratio is 26.4.

### 4.2. Experimental Setup

In this chapter, the key parameters of the proposed research and baseline system will be outlined. The experiment is based on a i7 6950x processor 3.0 GHz with 32GB and GTX1080 graphics card, which is implemented by reference to tensor flow. For each given parameter, there is a corresponding value and explanation in Table 4.

### 4.3. Experimental Results and Analysis

**Figure 6:**
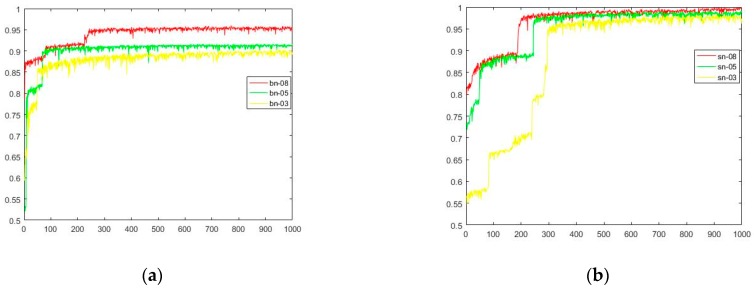

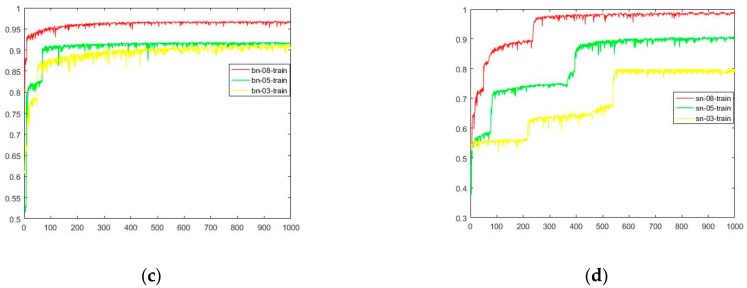
The result of BN-SSGAN and SN-SSGAN on training and test data set: (**a**) The results of BN-SSGAN under three different ratio conditions of labeled samples in all training samples, which are 0.3, 0.5 and 0.8, respectively; (**b**) The results of SN-SSGAN under three different radios conditions of labeled samples in all training samples, which are 0.3, 0.5 and 0.8 respectively; (**c**) The accuracy of BN-SSGAN under training data set; and (**d**) The accuracy of the SN-SSGAN under training data set.

The result of BN-SSGAN and SN-SSGAN on the test data set and the training data set are shown in Figure 6. The unit of the horizontal axis is 1000. There are four sub-graphs, which are listed as: (a) The results of BN-SSGAN under three different ratio conditions of labeled samples in all training samples, which are 0.3, 0.5 and 0.8, respectively; (b) The results of SN-SSGAN under three different radios conditions of labeled samples in all training samples, which are 0.3, 0.5 and 0.8 respectively; (c) The accuracy of BN-SSGAN under training data set; and (d) The accuracy of the SN-SSGAN under training data set.

From Figure 6a, it can be seen that the accuracy is only 0.95 when the ratio of labeled data in training data is 0.8. However, it can be seen from (b) that when there is a label data ratio of 0.3, the accuracy is already greater than 0.95. When it is 0.5 and 0.8, the results are very similar, that is, the label data of 0.5 can achieve a result of 0.8, this indicates that SSGAN reduces the dependence on labeled data. (c) and (d) are the corresponding accuracy rates of (a) and (b) on the training set, respectively. We find that (a) and (c) are almost similar, and the accuracy of (b) in the same iteration is higher than (d), which means that SSGAN has better generalization ability. With regard to the variance in accuracy, we found that SN-SSGAN is slightly better than BN-SSGAN.

In the following experiment designed to explore the effect of the ratio of labeled data, for a clearer explanation, see Table 5. Firstly, the ratio of all the training data to test data is 4:1, and the data set in the training set is randomly selected as 30%, 50% and 80% of the labeled data for supervised learning, and the rest is for unsupervised training. Also, the data from the unsupervised trainings includes fake data generated by the G. The test part is 20% randomly extracted from the whole dataset, and this part does not have any intersection with the training dataset. In this experiment, the ratio between fake data, real data and test data is 4:4:1. The ratio is between labeled and unlabeled data in the training data set.

Table 6 is the result of FFT+SVM, WDCNN, BN-SSGAN and SN-SSGAN based on 0.3, 0.5 and 0.8. 0.3, 0.5, and 0.8 are the ratios between real labeled data and real unlabeled data, respectively, which are explained in Table 5.

1. The traditional method of FFT+SVM does not work well. FFT+SVM and WDCNN are greatly affected by the amount of labeled data. When there is 50% labeled samples, WDCNN only reaches 94.59%, while SN-SSGAN reached 98.84%. When the proportion of labeled data is 80%, the accuracy of WDCNN reaches 98.79, while that of SN-SSGAN reaches 99.93%, which means SN-SSGAN is good at bearing fault diagnosis under the *G* spurring. Compared with BN-SSGAN, it can be clearly seen from this table that SN-SSGAN has obvious advantages in accuracy. SN-SSGAN reached 97.45% when the proportion of labeled data is 30%. Compared with BN-SSGAN, SN-SSGAN is able to guarantee a higher level of accuracy.
2. Although no unlabeled data is listed in Table 6, it is obvious that the lower the proportion of labeled data, the higher the proportion of unlabeled real data. Of course, the unlabeled data involved in training also includes fake data generated by the *G*. The number of fake data samples involved in training is related to the iterative proportion of the training process between the *G* and the *D*. In this algorithm, the ratio of samples trained between the *G* and the *D* is 1:1. Unlabeled real data plays a positive role in unsupervised learning, because even in the case of less labeled sample data, better results can still be obtained, which is enough to meet the demand for accuracy in the bearing diagnosis.
3. Fake data generated from the *G* of SN-SSGAN can solve the problem of insufficient data so that a good score is still obtained even when there is less data, although it takes more time for SN-SSGAN.

**Figure 7:**
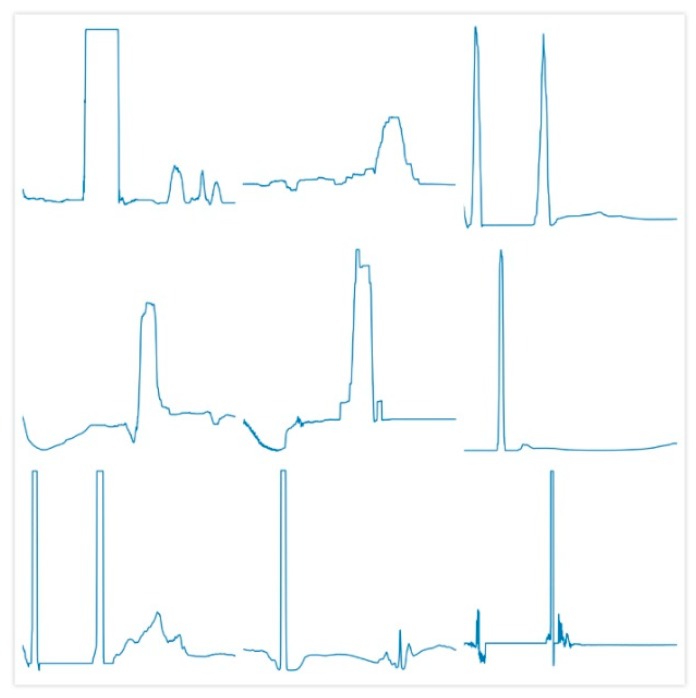
The fake data generated by the *G* of the SN-SSGAN.

The above reports all the results of the experiment, the following provides some visualization regarding fake data. For a naive generative model, researchers pay more attention to the quality of the generated data. For a discriminative model, researchers pay more attention to the discriminating ability of the model. Obviously, this paper belongs to the latter. Although for the former, when the training is over, the *D* or classifier will be discarded, and this paper only pays attention to promoting the training of the *D* with the help of the *G*, the visualization of fake data is still given, as shown in Figure 7, where there are 9 sub-graphs that show all of the fake data generated by *G*. The length of fake data is 2048. The first impression is similar to the time/frequency domain features processed by transformation of the time/frequency domain from raw vibration signals.

## 5. Conclusions

In this paper, an intelligent fault diagnosis method called semi-supervised GAN-based switchable normalization is proposed for fault diagnosis of rolling bearings. Novelties and contributions of this paper mainly include:

(1) Semi-supervised GAN is introduced to boost the accuracy of fault diagnosis under less labeled samples in training.
(2) Switchable normalization determines the appropriate normalization operation for each normalization layer in a deep network.

Through the above experimental validation, SSGAN based on switchable normalization (SN-SSGAN) ensures stable training and a good rate of accuracy. Also, by introducing switchable normalization, the model has stronger generalization ability. The *G* of SN-SSGAN in semi-supervised learning is more conducive to promoting the diagnosis accuracy. In SN-SSGAN, the adversarial process between the *G* and *D* facilitates the *D* to learn the latent features and make the most accurate judgment with a relatively low loss. This proposed method, which combines supervised and unsupervised learning in the training stage does not require post-fine tuning, so there is no additional operations required from storing knowledge in the training model to predicting with the model.

Though there are advantages in using the proposed method, the layers of *G* are considered to decrease. In exploring the adaptive update iterations, it is possible to temporarily decide whether to train the *D* or the *G*, or it is possible to look even farther. For example, the *G* needs to iterate 3 times and the *D* iterates once. This will be studied in our future work.

## Figures and Tables

**Table 1:** Operation name and output size of the generator.

No.	Operation Name	Output Size
1	FC, SN, ReLU	[BatchSize, 1, 1, 2048]
2	Deconv1, SN, ReLU	[BatchSize, 2, 1, 1024]
3	Deconv2, SN, ReLU	[BatchSize, 4, 1, 512]
4	Deconv3, SN, ReLU	[BatchSize, 8, 1, 256]
5	Deconv4, SN, ReLU	[BatchSize, 16, 1, 128]
6	Deconv5, SN, ReLU	[BatchSize, 32, 1, 64]
7	Deconv6, SN, ReLU	[BatchSize, 64, 1, 32]
8	Deconv7, SN, ReLU	[BatchSize, 128, 1, 16]
9	Deconv8, SN, ReLU	[BatchSize, 256, 1, 8]
10	Deconv9, SN, ReLU	[BatchSize, 512, 1, 4]
11	Deconv10, SN, ReLU	[BatchSize, 1024, 1, 2]
12	Deconv11, SN, ReLU	[BatchSize, 2048, 1, 1]
13	Deconv12, SN, tanh	[BatchSize, 2048, 1, 1]

**Table 2:** Operation name and output size of the discriminator.

No.	Operation Name	Output Size
1	Conv1, SN, LReLU	[BatchSize, 64, 1, 16]
2	Conv2, SN, LReLU	[BatchSize, 32, 1, 32]
3	Conv3, SN, LReLU	[BatchSize, 16, 1, 64]
4	Conv4, SN, LReLU	[BatchSize, 8, 1, 64]
5	Conv5, SN, LReLU	[BatchSize, 3, 1, 64]
6	FC, SN, LReLU	[BatchSize, 100]
7	Softmax	[BatchSize, K]

**Table 3:** Description of rolling element bearing data set.

Fault Location	Ball			Inner Race			Outer Race		Normal	
Fault diameter (inch)	0.007	0.014	0.021	0.007	0.014	0.021	0.007	0.014	0.021	0
Category labels	1	2	3	4	5	6	7	8	9	10
Training samples	1410	1410	1410	1410	1410	1410	1410	1410	1410	1410
Test samples	352	352	352	352	352	352	352	352	352	352

**Table 4:** The parameters and its explanation in the experiment.

Parameter	Value	Explanation
Batch size	32	Number of training samples at one time
Learning rate of *G*	0.0001	Generator’s learning rate
Learning rate of *D*	0.0001	Discriminator’s learning rate
Update rate	1	Assume update rate equals k, which indicates the discriminator updates k times and the generator updates one time.
Size of *z*	128	Random generated vector and the generator’s input
Optimizer of the *D*	Adam	(beta1 = 0.5)
Optimizer of the *G*	Adam	(beta1 = 0.5)
One-side label smoothing	0.9	Let label from 1/0 to 0.9/0.1 in unsupervised learning

In this paper, the contrasting experiment was based on the FFT+SVM and WDCNN [29]. Five convolution and pooling layers, one fully-connected layer and one Softmax classification are used in WDCNN.

**Table 5:** The ratio of fake data, labeled data, unlabeled data and test data when rate varied.

Ratio	Fake Data	Real Data		Test Data
		Labeled	Unlabeled	
0.3	4	1.2	2.8	1
0.5	4	2	2	1
0.8	4	3.2	0.8	1

**Table 6:** The result of four methods under different ratios of labeled data in training samples. Results are averaged over 10 seeds.

	The Ratio between Labeled Data and Unlabeled Data			Time (ms/signal)
	0.3	0.5	0.8	
FFT+SVM	68.36%	81.68%	85.13%	0.7
WDCNN	89.17%	94.59%	98.79%	0.28
BN-SSGAN	88.92%	91.15%	95.26%	0.31
SN-SSGAN	97.45%	98.84%	99.93%	0.39
